# Characterization of Clinical Phenotype to Glial Fibrillary Acidic Protein Concentrations in Alexander Disease

**DOI:** 10.1002/acn3.70305

**Published:** 2026-01-09

**Authors:** Amy T. Waldman, Asako Takanohashi, Joshua Y. Joung, Geraldine W. Liu, Kaley Arnold, Amy Pizzino, Walter Faig, Sarah Woidill, Sona Narula, Adeline L. Vanderver

**Affiliations:** ^1^ Division of Neurology Children's Hospital of Philadelphia Philadelphia Pennsylvania USA; ^2^ Department of Neurology Perelman School of Medicine at the University of Pennsylvania Philadelphia Pennsylvania USA; ^3^ Biostatistics and Data Management Core Children's Hospital of Philadelphia Philadelphia Pennsylvania USA

**Keywords:** Alexander disease, biomarker, GFAP, leukodystrophy

## Abstract

**Objective:**

To determine the concentration of glial fibrillary acidic protein (GFAP) in cerebrospinal fluid (CSF) and plasma in Alexander disease (AxD) and whether GFAP levels are predictive of disease phenotypes.

**Methods:**

CSF and plasma were collected (longitudinally when available) from AxD participants and non‐AxD controls. The concentration of GFAP was compared between groups using Wilcoxon rank sum test. The Kruskal‐Wallis test was used to compare GFAP concentrations across multiple groups (clinical phenotype or common genetic variants occurring at p.Arg79, p.Arg88, p.Arg239).

**Results:**

GFAP concentrations were significantly elevated at baseline in both AxD CSF (*N* = 44) compared to controls (*N* = 46, *p* < 0.0001) and AxD plasma (*N* = 96) compared to other leukodystrophy plasma controls (*N* = 67, *p* < 0.0001). CSF and plasma GFAP concentrations differed between AxD cerebral, intermediate, and bulbospinal phenotypes (Kruskal‐Wallis test, CSF: *p* = 0.0235, plasma: *p* < 0.0001). The median GFAP change/year among patients sampled < 8 years of age at baseline was an increase of 91,100 [pg/mL]/year (IQR 136,050) in CSF and 2850 [pg/mL]/year (IQR 9500) in plasma compared to patients > 8 years whose medians decreased over time (−41,000 [pg/mL]/year [IQR 90,300], CSF; −843 [pg/mL]/year [IQR 3900], plasma). The fold change in GFAP from first to last sampling were significantly different before and after 8 years at baseline (Wilcoxon rank sum test, CSF: *p* = 0.0232, plasma: *p* = 0.0002). A significant association was not detected between *GFAP* variant and GFAP levels.

**Interpretation:**

GFAP concentrations are higher in the cerebral phenotype and increase over time in young children. These data can be used to formulate biomarker qualification and context‐of‐use in AxD.

## 1 Introduction

Alexander disease (AxD) is caused by toxic gain of function pathologic variants in the *GFAP* gene leading to the accumulation of glial fibrillary acidic protein (GFAP) in astrocytes [1, 2]. Elevated GFAP leads to the formation of Rosenthal fibers in the brain and spinal cord, followed by a cellular stress response and reactive astrogliosis [3]. Since current therapeutic strategies for AxD target GFAP reduction [4], there is an urgent need to further understand how GFAP levels in accessible fluids, such as CSF and blood, relate to the clinical phenotype and standardize the protocols for GFAP assays.

Prior studies have demonstrated elevations of GFAP in CSF and plasma of AxD subjects using ELISA [5, 6]. Kyllerman et al. studied three individuals, each with a different GFAP variant, who were ages 9–24 years at the times of sample collection [5]. All 3 had CSF values that were significantly above the reference range. Subsequently, Jany et al. examined GFAP in CSF and blood using samples from a much larger cohort [6]. In CSF, Jany confirmed the elevation in AxD compared to controls but still had too few samples to further delineate potential connections to subtypes of the clinical phenotype. In blood, Jany found significantly elevated values in those with infantile and juvenile onset. In contrast, adult‐onset individuals had blood values that were indistinguishable from controls. Ashton et al. repeated the analysis on a different testing platform and did show differences between adult and control subjects [7]. However, neither Jany nor Ashton had sufficient clinical information to further investigate the potential relationships to the newer systems for patient classification as have been proposed by Prust and Yoshida [8, 9]. In addition, sampling for each patient occurred only once; thus, change over time could not be assessed.

With a growing focus on neurologic biomarkers, there has been tremendous interest in GFAP as a measure of astrocyte stress response. Increased GFAP concentrations occur in several conditions such as traumatic brain injury, multiple sclerosis, and Alzheimer's disease [10, 11]. In addition, technical advances in assay design and instrumentation have increased the sensitivity and dynamic range of the measurements. Despite the wide application of GFAP in other areas of neuroscience, there is an unmet need regarding GFAP biomarker method validation and fit‐for‐purpose in AxD.

The objective of the current study was to determine GFAP concentrations in a prospective natural history study of AxD patients compared to controls using the commercially available Simoa‐based assay. We further sought to explore the potential context of use for the measurement of GFAP as a biomarker in AxD, including the various factors that may influence GFAP levels such as age, genetic variant, and disease phenotype. Finally, we examined GFAP concentrations at multiple time points. Together, these experiments represent an initial step in understanding the use of GFAP as a biomarker in AxD and advancing our understanding of the variability associated with the measurement.

## 2 Methods

### 2.1 Cohort

Patients with clinical and imaging features of AxD, confirmed by genetic analysis of *GFAP*, were enrolled in a natural history study entitled “Evaluation of Outcome Metrics in Alexander disease” (ClinicalTrials.gov identifier: NCT02714764). The study, which began in January 2016, was designed to capture prospective natural history data and both clinician‐ and patient‐ reported outcomes. Beginning in the middle of 2017, subjects were also invited to participate in an optional sub‐study, which included the collection of CSF and blood samples. To minimize risk, only 2 lumbar punctures, performed 12 months apart, were permitted by the Institutional Review Board if obtained solely for research purposes. However, our protocol also allowed us to collect CSF from a clinically indicated lumbar puncture. As such, additional CSF was available for some patients who underwent clinical lumbar punctures. Additional AxD subjects were identified from the Myelin Disorders Biorepository Project (ClinicalTrials.gov identifier: NCT03047369) over the same time period. The AxD sub‐cohort contained 2 sets of same‐sex twins as well as a family with 3 affected siblings.

CSF control samples were obtained from left‐over clinical samples from patients who underwent lumbar punctures for other indications such as malignancy surveillance or administration of chemotherapy. These samples were available within the Myelin Disorders Biorepository Project. As these samples were obtained from a clinical lab, additional information on the subjects' symptoms or outcomes was not available. Plasma controls were selected from individuals with hypomyelinating disorders represented within the Myelin Disorders Biorepository Project, as these disorders were less likely to involve primary astrocyte pathology. For both CSF and plasma controls, samples were age‐matched to the AxD cohort with respect to age at sample collection with a maximum discrepancy of 1 year. Longitudinal samples were not available for controls.

### 2.2 Sample Processing

CSF was collected in polypropylene tubes and immediately placed in a wet ice bath for transport to the research laboratory for processing. CSF was then transferred into a new, clean 15 mL conical tube and spun at 300 x g (rcf) for 10 min at 4°C. Without disrupting the small invisible pellet of CSF cells, the supernatant was transferred into a clean 15 mL conical tube and then aliquoted (250 μL) into protein low‐binding microtubes and stored at −80°C. Plasma samples were collected as whole blood in EDTA collection tubes and processed within 48 h. EDTA whole blood was transferred to a 15 mL conical tube and centrifuged at 2000 x g for 5 min at room temperature; plasma was then aliquoted into protein low‐binding microtubes and stored at −80°C.

### 2.3 Phenotyping

AxD patients were classified according to the 3 published systems [8, 9, 12]. The age‐based system was defined by age at neurologic symptom onset (neonatal < 30 days, infantile: 31 days–< 2 years; juvenile: 2–< 13 years, adult: ≥ 13 years) [13, 14]. The Prust classification system was also used (Type I vs. Type II) [8]. Finally, patients were classified according to the Yoshida system [9] and defined as cerebral, intermediate, or bulbospinal. Although the intermediate phenotype is typically defined as having clinical and imaging features of both cerebral and bulbospinal forms, the criteria for this classification in our cohort were loosened to include subjects with either clinical or imaging features of both cerebral and bulbospinal disease. Cerebral subjects were further divided into neonatal (< 30 days) vs. infantile onset.

### 2.4 Genetic Variants

Variants in *GFAP* were aligned with the MANE transcript based on the transcript reported in the testing laboratory report, if available. For cases with no report or no transcript listed, the variant was checked using the date of variant discovery if known, NCBI/Ensembl, and a comparison of nucleotide and amino acid changes to identify the most likely transcript.

### 2.5 Simoa GFAP Assay for CSF and Plasma

GFAP protein levels were measured from CSF and plasma samples using an ultrasensitive Single Molecule Array (Simoa GFAP Discovery Kit, Quanterix, #102336). The GFAP immunoassays were performed using the manufacturer's standardization controls and samples were tested in duplicate. The concentration of GFAP in each well was averaged and then multiplied by the dilution factor to provide a single (mean) GFAP concentration for each sample. The coefficient of variation (CV) was calculated to verify data integrity, including intra‐ and inter‐assay variability, for each sample's mean concentration. A sensitivity analysis was performed excluding samples with high intra‐assay variability defined as a CV > 10% in order to assess a change in the significance of results. Finally, an experiment to evaluate variability in CSF aliquots (two stored vials from a single lumbar puncture) was performed using 6 samples with 4 aliquots (*N* = 24) tested within plate (2) and between plates (2), all performed in duplicate.

Serial dilutions of the manufacturer's recommended dilution factors were utilized to identify an optimal dilution that minimized ceiling effects (results that were above the limit of quantification). In addition, several aliquots were subjected to multiple freeze–thaw cycles to test sample integrity. Only first thaw samples were used for the final data set.

### 2.6 Statistical Analysis

Descriptive statistics, including median, range, IQR, and proportions, were used to capture clinical features of AxD participants and control groups. Baseline age at sample collection, sex distribution, distributions of clinical features, and GFAP concentration were compared using chi‐squared test for discrete variables and Wilcoxon rank sum test or Kruskal‐Wallis test for continuous variables compared across more than 2 groups. Repeated measures correlation (rmcorr) was used to assess correlation between matched CSF and plasma samples as some subjects had repeated collections of matched samples. Longitudinal data was explored using Loess regression and Wilcoxon rank sum test. Analyses and figures were generated in R studio [15].

## 3 Results

### 3.1 Patient Enrollment

**FIGURE 1:**
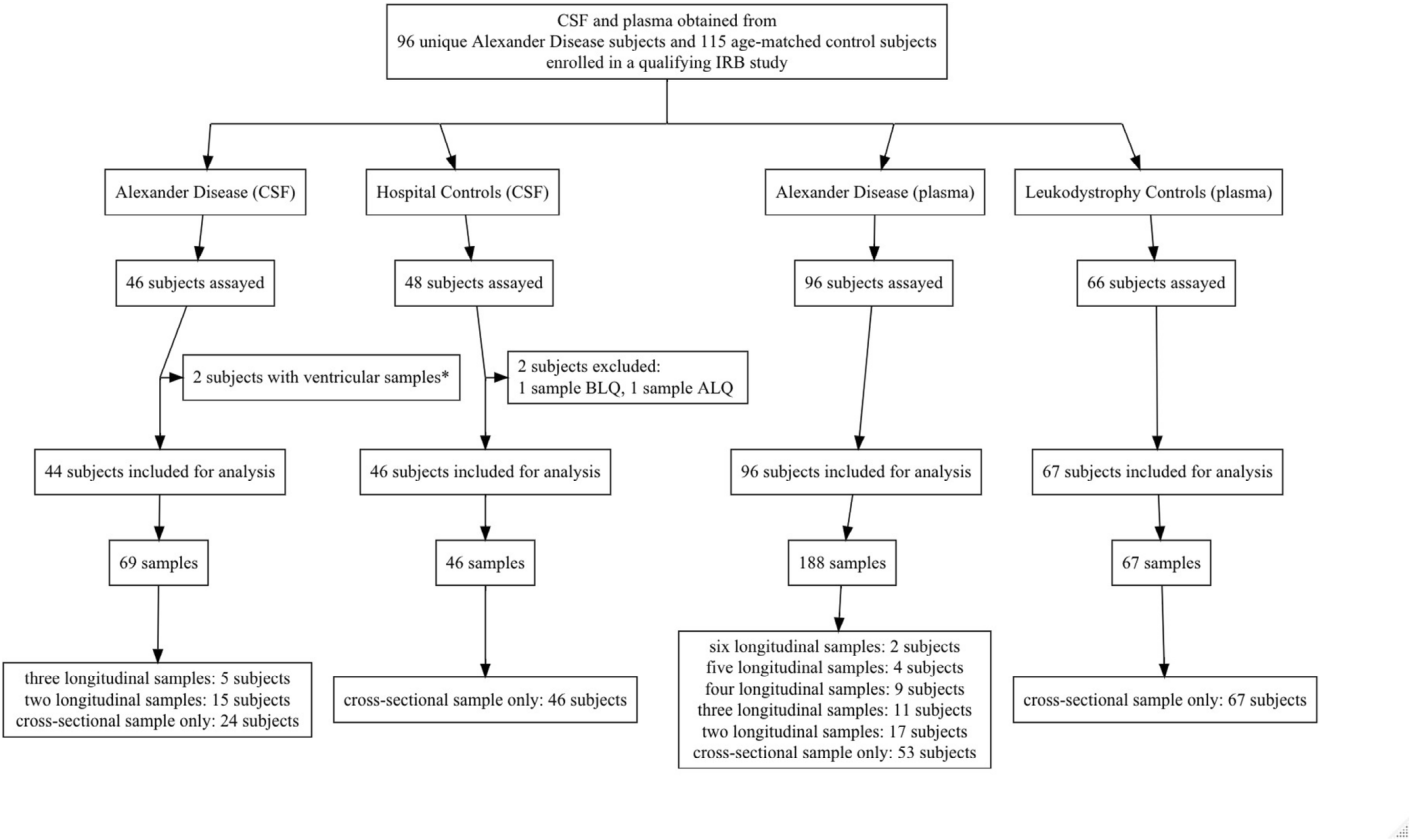
Subject enrollment and sample collection.

Ninety‐six AxD participants provided plasma samples, 46 of whom also provided concurrent CSF (Figure 1, Table 1). Two subjects had CSF collected from ventricular fluid during neurosurgical procedures; these are shown in visualizations only. Given the limited data for those with ventricular samples, these data points were excluded from further analyses. The final AxD cohort consisted of plasma samples from 96 participants, of whom 44 also provided concurrent CSF samples. Longitudinal plasma samples were available for 43 subjects and longitudinal CSF samples were available for 20 subjects. The median time between longitudinal samplings was 1.6 years for CSF (IQR 1.7 years, range 0.5–5.9 years) and 1.2 years for plasma (IQR 0.68 years, range 0.1–14.1 years). Concordant longitudinal samples were available for 20 subjects with a median of 2 instances (range 1–3 longitudinal samples). Control CSF was collected from residual fluid from lumbar punctures performed for the following reasons: acute lymphoblastic leukemia, acute myeloid leukemia, administration of chemotherapy, and shunt malfunctions. Plasma controls were collected from 67 subjects with hypomyelinating leukodystrophies: 18 with Pelizaeus‐Merzbacher disease (PMD), 22 with POLR3‐related leukodystrophy, and 27 with *TUBB4A*‐related leukodystrophy. Age at sample collection and patient demographics are presented in Table 1. For the cohort, the median time from onset at baseline sampling was 4.7 years (IQR 7.1 years, range 0.30–38.1 years).

**TABLE 1:** Cohort description at baseline.

	Lumbar CSF			EDTA plasma		
	Alexander disease	Hospital controls	*p*	Alexander disease	Leukodystrophy controls	*p*
	*N* = 44	*N* = 46		*N* = 96	*N* = 67	
*Sex (%)*						
Female	20 (45.5%)	18 (39.1%)	0.6938	43 (44.8%)	24 (35.8%)	0.3258
Male	24 (54.5%)	28 (60.9%)		53 (55.2%)	43 (64.2%)	
*Race/ethnicity*						
Asian	1 (2.3%)	NA	NA	3 (3.1%)	5 (7.5%)	0.6529
Bi‐/Multiracial	1 (2.3%)	NA		3 (3.1%)	4 (6.0%)	
Black/African American	0 (0.0%)	NA		4 (4.2%)	3 (4.5%)	
Caucasian/White	37 (84.1%)	NA		71 (74.0%)	41 (61.2%)	
Hispanic/Latino	0 (0.0%)	NA		2 (2.1%)	3 (4.5%)	
Other	1 (2.3%)	NA		1 (1.0%)	1 (1.5%)	
Unknown/not reported	4 (9.1%)	NA		12 (12.5%)	10 (14.9%)	
*Age at sampling (years)*						
Median (Q1; Q3)	7.17 (3.56; 11.7)	7.00 (4.00; 12.0)	0.9100	8.98 (2.97; 19.2)	9.34 (4.59; 15.9)	0.7990
Range [min; max]	[0.97; 30.8]	[0.830; 18.0]		[0.10; 77.2]	[0.71; 42.6]	
*Baseline GFAP (pg/mL)*						
Median (Q1; Q3)	303,000 (122,000; 507,000)	3720 (2520; 5930)	< 0.0001	15,400 (4460; 26,800)	276 (206; 472)	< 0.0001
Range [min; max]	[48,300; 1,070,000]	[33.7; 19,100]		[345; 103,000]	[46.1; 910]	

### 3.2 Assay Validation

Several experiments were performed to validate use of the Simoa assay for AxD (detailed in the Supporting Information). Dilution factors of 1:40 for CSF and 1:4 for plasma were utilized for the initial experiments based on the manufacturer's data sheet (minimum required dilution; however, at these dilutions in AxD patients, the GFAP concentrations were frequently above the limit of detection). Through additional experiments, dilution factors of 1:1600 for CSF and 1:160 for plasma in AxD subjects were determined to provide optimal results (Figure S1). Using these dilutions, all AxD CSF and plasma samples were within the limits of assay detection (resulted in no missing values, Table S1). For all AxD samples, including longitudinal data to demonstrate the reliability of the assay, the range of GFAP concentration in CSF at 1:1600 dilution was 48,300 to 1,070,000 pg/mL compared to non‐leukodystrophy controls tested at 1:40 dilution with a range of 33.7 to 19,100 pg/mL. Similarly, the range of plasma GFAP concentration at a dilution of 1:160 in AxD was 345 to 103,000 pg/mL, compared to a dilution of 1:4 in leukodystrophy controls whose range was 46.1 to 910 pg/mL.

Freeze–thaw experiments were performed to determine the stability of GFAP in CSF in 5 samples across the range of GFAP levels in AxD (Figure S2, Table S2). The greatest change in GFAP occurred after the first thaw with a mean fold change of 0.54 or a reduction of 46%. After 4 thaws, the sample concentration was 20% (range 15%–26%) of the original sample concentration on average. Between each additional thaw, the average fold change lessened after each additional thaw by 0.57‐fold after the second thaw, 0.87‐fold after the third thaw, and 0.88‐fold after the fourth thaw. Only one freeze thaw cycle was tested in plasma which showed that GFAP concentration remained mostly stable.

Intra‐assay variability (running duplicate samples on the same plate) was explored through the CV, which was < 10% at all dilutions (Table S3). Across all sample groups, the mean CV was 2.62% (range 0.13%–23.06%) in CSF and 4.33% (range 0.01%–34.53%) in plasma (Table S3). Inter‐assay variability was explored using first thaw samples at the same laboratory on plates purchased at the same time but run several months apart. Due to limited availability of CSF samples, no hospital control samples were available for control inter‐assay variability and few AxD samples were run serially. Overall, the median CV across runs was 13.86% (range 2.41%–35.38%) in CSF (AxD: *N* = 13) and 6.55% (range 0.25%–32.48%) in plasma (AxD: *N* = 45, leukodystrophy control: *N* = 10; Table S4). Samples with a CV of > 10% (*N* = 2, 2.2% CSF samples and *N* = 33, 20.2% plasma samples, *N* = 9 single replicate value) were excluded during a sensitivity analysis to determine if these samples impacted the significance of the results; the significance remained unchanged with the exclusion of these samples (Table S5).

Finally, aliquot (including additional intra‐assay and inter‐assay) variability in CSF was explored in a controlled experiment (Figure S3A–C). Replicate concentrations ranged from 0.0%–8.0% CV% with a mean difference of 1.9 pg/mL between replicates (Figure S3A). Mean aliquot concentrations within the same plate were not significantly different (Wilcoxon signed rank test: plate 1, *p* = 0.0625; plate 2, *p* = 0.688), and the CV% ranged from 1.05%–15.9% (Figure S3B). Mean concentrations of the sample (4 values) within each plate were not significantly different between plates (Wilcoxon signed rank test: *p* = 0.438) and CV% ranged from 3.6% to 16.2% (Figure S3C).

### 3.3 GFAP Concentrations in CSF and Plasma

**FIGURE 2:**
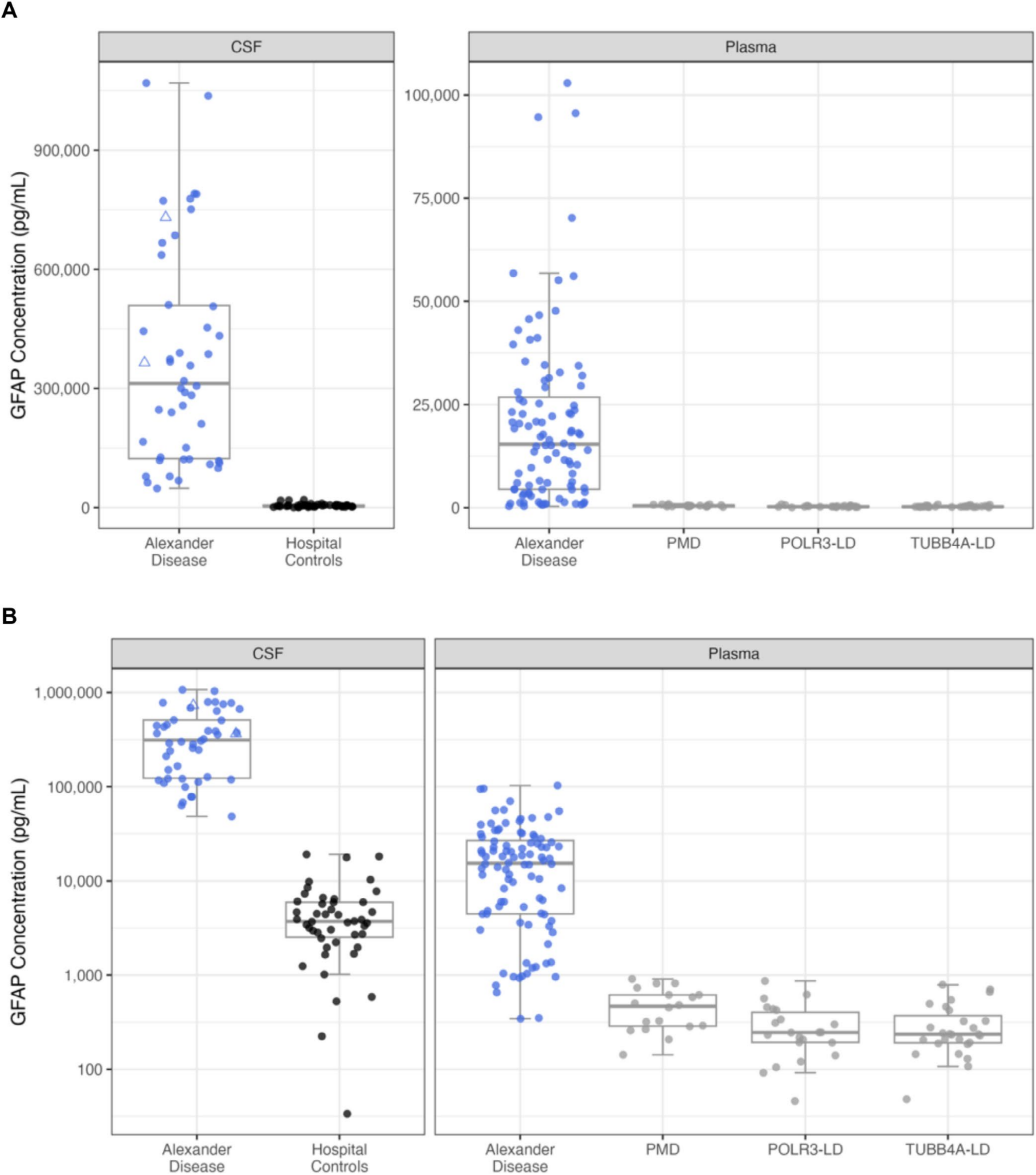
Baseline GFAP in Alexander disease (AxD) and control CSF and plasma. Median (IQR) baseline GFAP concentration in lumbar CSF was 303,000 pg/mL (385,000 pg/mL) in AxD and 3720 pg/mL (3410 pg/mL) in hospitalized controls. Median (IQR) baseline GFAP concentration in plasma was 15,400 pg/mL (22,340 pg/mL) in AxD and 276 pg/mL (266 pg/mL) in hypomyelinating‐leukodystrophy controls. GFAP was significantly higher in AxD compared to controls in both CSF and plasma (Wilcoxon rank sum test, both *p* < 0.0001). Ventricular CSF samples are designated by a triangle. (A) Mean GFAP concentration in raw scale. (B) Mean GFAP concentration on log‐scale.

**FIGURE 3:**
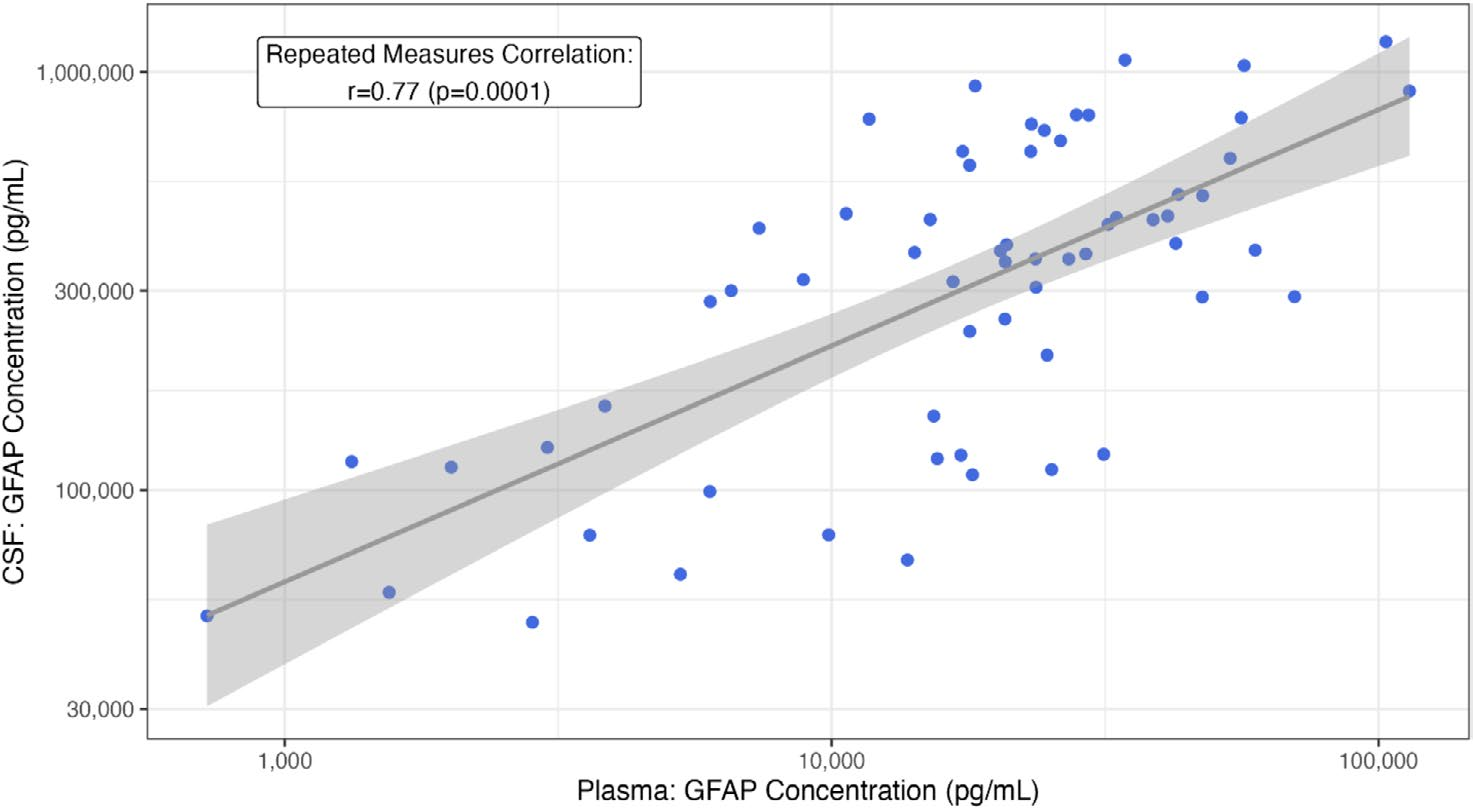
Correlation of GFAP concentration in paired CSF and plasma samples. CSF and plasma GFAP concentrations in contemporaneously obtained samples are strongly and positively correlated (Spearman correlation *r* = 0.77, *p* = 0.0001).

Baseline CSF GFAP concentrations of AxD participants were significantly elevated compared to those of age‐matched hospital controls (Wilcoxon rank sum test, *p* < 0.0001; Figure 2, Table 1). Similarly, plasma GFAP concentrations in AxD participants were also significantly elevated compared to those of other hypomyelinating leukodystrophies (Wilcoxon rank sum test, *p* < 0.0001; Figure 2, Table 1). Among AxD participants, CSF GFAP levels were positively correlated with plasma GFAP levels (Repeated measures correlation, *r* = 0.77, *p* = 0.0001; Figure 3).

**FIGURE 4:**
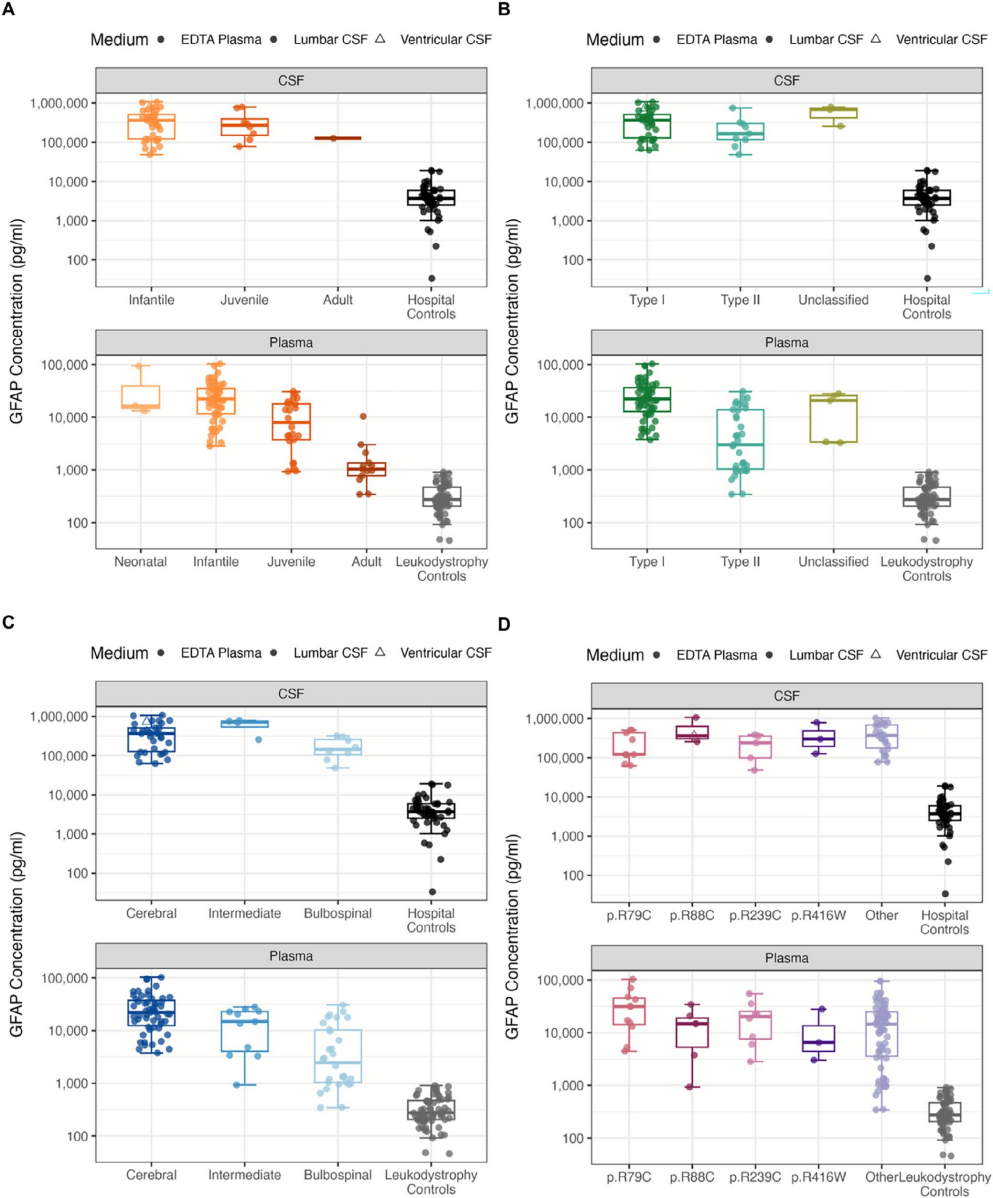
GFAP concentration as a function of disease phenotypes in CSF and plasma. CSF and plasma GFAP concentrations by AxD phenotype classifications. (A) GFAP concentrations were not significantly different between onset categories in CSF but were in plasma (Kruskal‐Wallis test: CSF, *p* = 0.7231; plasma, *p* < 0.0001). In plasma, GFAP levels were significantly different between the infantile group and juvenile or adult (Dunn's test for pairwise comparisons: *p* = 0.0019 and *p* < 0.0001, respectively), juvenile and adult (*p* = 0.0193), neonatal and adult (*p* = 0.0170). The neonatal group did not significantly differ from infantile or juvenile groups (*p* = 0.9681 and *p* = 0.2909, respectively) but comprised a very small group size (*N* = 3). (B) Excluding unclassified samples, GFAP concentrations were not significantly different between Type I and Type II in CSF but were significantly different in plasma (Kruskal‐Wallis test: CSF, *p* = 0.1280; plasma, *p* < 0.0001). (C) CSF GFAP concentrations were significantly different between cerebral, intermediate, and bulbospinal patients (Kruskal‐Wallis test: CSF, *p* = 0.0235; plasma, *p* < 0.0001). In CSF, intermediate samples were significantly higher than bulbospinal samples; while in plasma cerebral samples were significantly higher than bulbospinal samples (Dunn's test for pairwise comparisons: *P* = 0.0250 and *p* < 0.0001, respectively). (D) Variant classifications were not significantly different in CSF or plasma GFAP concentrations (Kruskal‐Wallis test: CSF, *p* = 0.2690; plasma: *P* = 0.3130). Significance of these results remained the same when the samples with high variability (CV > 10%) were excluded in a sensitivity analysis.

Clinical features including age of onset, Prust and Yoshida classifications, and *GFAP* variants hypothesized to contribute to GFAP variation were further explored. AxD patients were separated by disease phenotype (Figure 4). Significant differences in baseline CSF GFAP concentration were observed only when classifying participants by cerebral, intermediate, and bulbospinal phenotypes (*p* = 0.0235, Figure 4C); significantly higher GFAP concentrations were seen in the intermediate classification (*N* = 4) compared to the bulbospinal classification (*N* = 8, Dunn's test for pairwise comparisons: *p* = 0.0250). Baseline CSF GFAP concentrations did not differ between subjects classified as Type I vs. Type II phenotypes (*p* = 0.1280, Figure 4B) or the age‐of‐onset based groups (*p* = 0.7231, Figure 4A). It should be noted that, in CSF, the sample sizes were smaller. Additionally, some patients had overlapping features of Type I and Type II disease and were thus grouped as “unclassified” (Figure 4B). In plasma, GFAP levels were significantly different between categories according to all phenotyping systems (all *p* < 0.0001, Figure 4A–C). We also explored GFAP concentrations among subjects with common pathogenic variants in *GFAP* (Table 2, Table 3). GFAP concentrations did not differ significantly in CSF or plasma between subjects with p.Arg79, p.Arg88, p.Arg239, or p.Arg416 missense variants (Kruskal‐Wallis test: CSF: *p* = 0.2690, plasma: *p* = 0.3130; Figure 4D). The distributions of GFAP concentrations and age at sampling for additional variants (with *N* > 1) are shown in Figure S4A,B, respectively. The complete list of included variants within the AxD cohort can be found in Table S6.

**TABLE 2:** Clinical features in association with Yoshida classification and baseline CSF GFAP.

	Lumbar CSF			
	Cerebral	Intermediate	Bulbospinal	*p*
	*N* = 32	*N* = 4	*N* = 8	
*Baseline GFAP (pg/mL)*				
Median (Q1; Q3)	362,000 (122,000; 507,000)	718,000 (578,000; 761,000)	146,000 (107,000; 260,000)	0.0235
Range [min; max]	[63,000; 1,070,000]	[257,000; 790,000]	[48,300; 319,000]	
*Baseline age (years)*				
Median (Q1; Q3)	4.55 (2.58; 7.67)	10.2 (8.99; 11.5)	16.8 (14.0; 22.2)	< 0.0001
Range [min; max]	[0.97; 19.3]	[7.20; 13.7]	[9.98; 30.8]	
*Variant classification (%)*				
p.Arg79Cys	9 (28.1%)	0 (0%)	0 (0%)	0.0450
p.Arg88Cys	1 (3.1%)	1 (25.0%)	0 (0%)	
p.Arg239Cys	4 (12.5%)	0 (0%)	1 (12.5%)	
p.Arg416Trp	0 (0%)	1 (25.0%)	2 (25.0%)	
Other	18 (56.3%)	2 (50.0%)	5 (62.5%)	
*Prust classification (%)*				
Type I	32 (100%)	0 (0%)	0 (0%)	0.0005
Type II	0 (0%)	1 (25.0%)	8 (100%)	
Unclassified	0 (0%)	3 (75.0%)	0 (0%)	
*Onset classification (%)*				
Neonatal	0 (0%)	0 (0%)	0 (0%)	0.0005
Infantile	32 (100%)	2 (50.0%)	1 (12.5%)	
Juvenile	0 (0%)	2 (50.0%)	6 (75.0%)	
Adult	0 (0%)	0 (0%)	1 (12.5%)	

**TABLE 3:** Clinical features in association with Yoshida classification and baseline plasma GFAP.

	EDTA plasma			
	Cerebral	Intermediate	Bulbospinal	*p*
	*N* = 55	*N* = 11	*N* = 30	
*Baseline GFAP (pg/mL)*				
Median (Q1; Q3)	22,100 (12,500; 37,500)	14,800 (4100; 23,000)	2490 (1040; 10,200)	< 0.0001
Range [min; max]	[3760; 103,000]	[931; 28,000]	[345; 30,800]	
*Baseline age (years)*				
Median (Q1; Q3)	3.75 (2.22; 7.80)	11.9 (9.35; 25.1)	22.1 (13.9; 53.1)	< 0.0001
Range [min; max]	[0.10; 26.2]	[2.36; 32.3]	[8.06; 77.2]	
*Variant classification (%)*				
p.Arg79Cys	11 (20.0%)	0 (0%)	0 (0%)	0.0015
p.Arg88Cys	3 (5.5%)	3 (27.3%)	0 (0%)	
p.Arg239Cys	7 (12.7%)	0 (0%)	1 (3.3%)	
p.Arg416Trp	0 (0%)	1 (9.1%)	2 (6.7%)	
Other	34 (61.8%)	7 (63.6%)	27 (90.0%)	
*Prust classification (%)*				
Type I	55 (100%)	1 (9.1%)	0 (0%)	0.0005
Type II	0 (0%)	5 (45.5%)	30 (100%)	
Unclassified	0 (0%)	5 (45.5%)	0 (0%)	
*Onset classification (%)*				
Neonatal	3 (5.5%)	0 (0%)	0 (0%)	0.0005
Infantile	50 (90.9%)	5 (45.5%)	1 (3.3%)	
Juvenile	2 (3.6%)	6 (54.5%)	16 (53.3%)	
Adult	0 (0%)	0 (0%)	13 (43.3%)	

**FIGURE 5:**
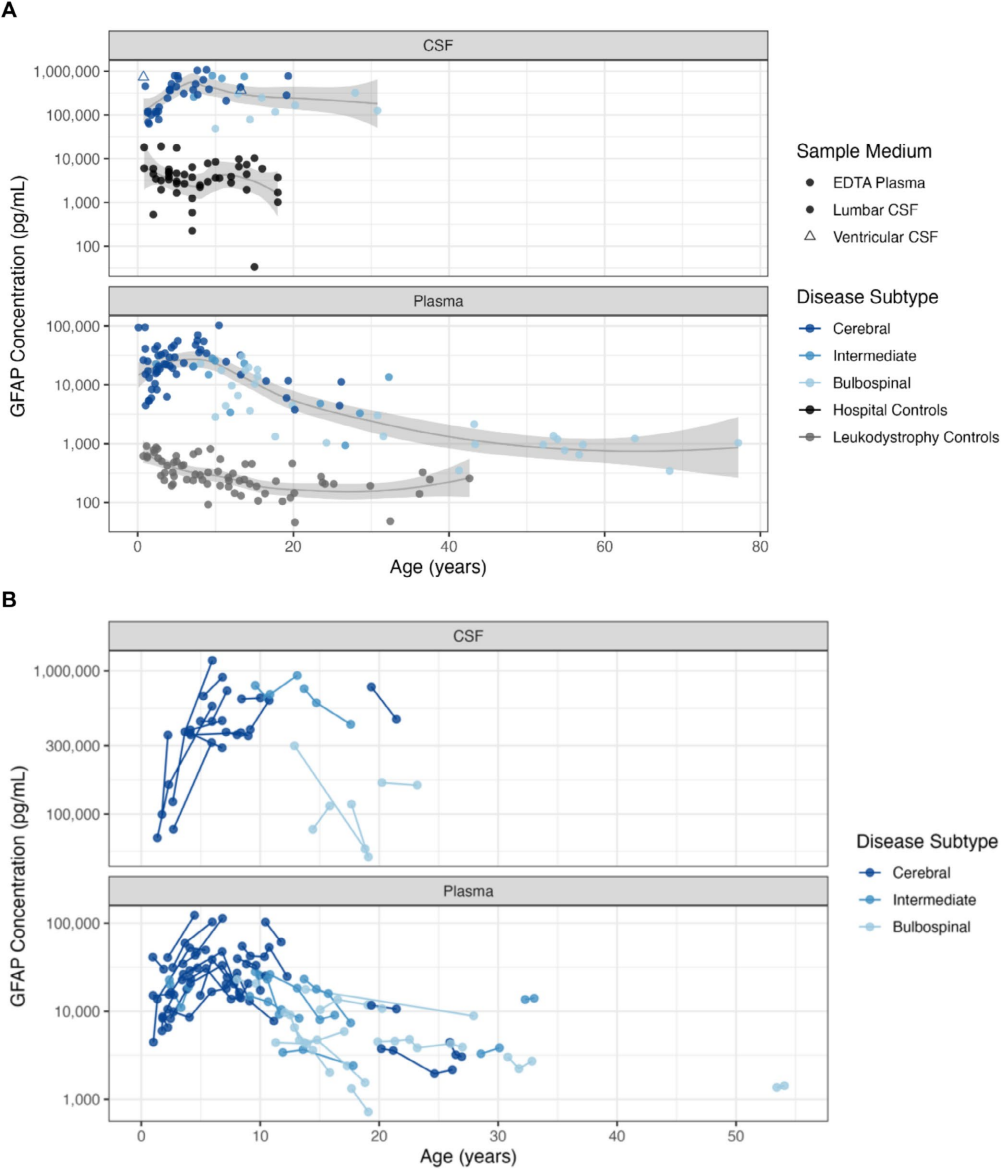
GFAP concentrations as a function of age at sample collection in CSF and plasma. (A) Cross‐sectional baseline GFAP by sample age. LOESS peak GFAP values in AxD subjects at baseline were 8.2 years in CSF and 7.9 years in plasma. (B) Subjects with longitudinal samples over time shown by Yoshida classification.

We then explored GFAP concentration as a function of age in the control population (cross sectional data only). For CSF, no relationship between age and GFAP concentration was noted (*r* = −0.12, *p* = 0.4236; Figure 5). However, in plasma of the leukodystrophy controls, there was a negative association between GFAP concentration and age (*r* = −0.63, *p* < 0.0001; Figure 5A).

Next, we explored the potential confounding effect of age on association of GFAP concentration and disease subtype in AxD. Figure 5A shows GFAP concentration as a function of age and Yoshida phenotype for baseline GFAP levels. Loess regression was used to determine the peak GFAP concentration using cross sectional baseline data (8.2 years for CSF and 7.9 years for plasma). Nearly all subjects sampled at baseline age prior to 8 years fell into the cerebral classification (CSF: 100%, plasma: 94.4%); thus, prior to 8 years at baseline sampling, we could not assess the impact of age independent of disease classification. As a sensitivity analysis, we examined the subset of baseline data after 8 years where all the phenotypes were represented. The sample age distribution for patients over age 8 years was similar across Yoshida classification groups (Kruskal‐Wallis test, CSF: *p* = 0.231, plasma: *p* = 0.538), but the GFAP concentrations were significantly different across the 3 phenotypes in plasma and CSF (Kruskal‐Wallis test, CSF: *p* = 0.0302, plasma: *p* = 0.0486).

**FIGURE 6:**
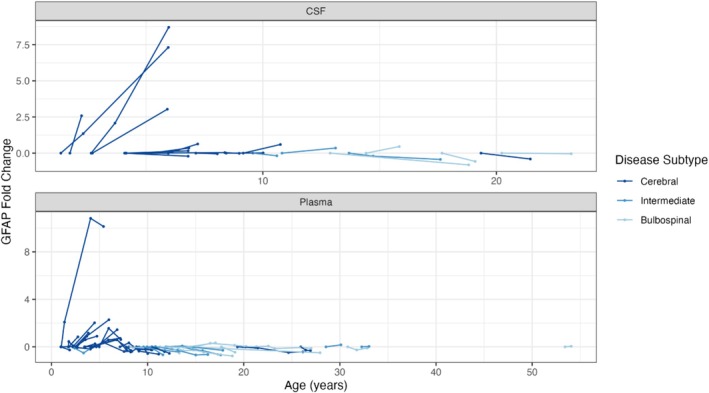
Fold change in GFAP concentration by age at sample collection. The change in GFAP concentration, normalized to each individual's baseline value, is shown as a function of age at sampling and Yoshida classification. Fold change was significantly different between participants first sampled prior to 8 years and after 8 years in both CSF and plasma (Wilcoxon rank sum test: *p* = 0.0140 and *p* < 0.0001, respectively).

Next the change in GFAP concentration over time was explored using longitudinal sampling data (> 1 sample collection). Longitudinal CSF and plasma data were available for 20 and 43 AxD participants, respectively (Figure 1). Longitudinal data by age at collection and Yoshida subtype is shown in Figure 5B. To further assess trends in GFAP levels between timepoints, the fold change for individual patients was measured and plotted as a function of age in years (Figure 6). Based on the peak of the Loess function, change was compared dichotomously before and after 8 years baseline age. Overall change during the follow‐up period is described in Table 4. Overall fold change was significantly different between subjects first sampled prior to 8 years and after 8 years in both CSF and plasma (Wilcoxon rank sum test: *p* = 0.0232 and *p* = 0.0002, respectively). In the subjects sampled prior to 8 years at baseline, the median change in GFAP was an increase of 91,100 [pg/mL]/year (IQR 136,050 [pg/mL]/year) in CSF and 2850 [pg/mL]/year (IQR 9500 [pg/mL]/year) in plasma (Table 4). In the subjects sampled after 8 years at baseline, the median change in GFAP was −41,000 [pg/mL]/year (IQR 90,300 [pg/mL]/year) in CSF and −843 [pg/mL]/year (IQR 3899 [pg/mL]/year) in plasma (Table 4).

**TABLE 4:** GFAP change from baseline in longitudinal CSF and plasma.

	Lumbar CSF			EDTA plasma		
	< 8 years at baseline	≥ 8 years at baseline	Overall	< 8 years at baseline	≥ 8 years at baseline	Overall
	*N* = 10	*N* = 10	*N* = 20	*N* = 18	*N* = 25	*N* = 43
*Baseline GFAP concentration (pg/mL)*						
Median (Q1; Q3)	362,000 (105,000; 384,000)	513,000 (199,000; 735,000)	371,000 (121,000; 644,000)	20,600 (11,700; 30,900)	10,400 (3760; 23,200)	15,100 (5250; 26,800)
Range [min; max]	[68,100; 667,000]	[78,100; 790,000]	[68,100; 790,000]	[4460; 46,700]	[1330; 103,000]	[1330; 103,000]
*Last GFAP concentration (pg/mL)*						
Median (Q1; Q3)	403,000 (353,000; 686,000)	441,000 (125,000; 640,000)	436,000 (309,000; 646,000)	19,800 (15,700; 40,300)	7370 (2720; 10,700)	14,100 (5300; 22,600)
Range [min; max]	[290,000; 1,180,000]	[50,100; 927,000]	[50,100; 1,180,000]	[6560; 123,000]	[721; 61,200]	[721; 123,000]
*GFAP concentration difference (pg/mL)*						
Median (Q1; Q3)	236,000 (11,300; 275,000)	−36,900 (−218,000; 29,200)	22,900 (−69,700; 238,000)	7280 (−3480; 13,500)	−1600 (−9110; −591)	−1080 (−7680; 1030)
Range [min; max]	[−77,700; 1,060,000]	[−328,000; 242,000]	[−328,000; 1,060,000]	[−12,600; 82,400]	[−41,700; 1480]	[−41,700; 82,400]
*Time from baseline (years)*						
Median (Q1; Q3)	2.69 (1.77; 3.29)	1.85 (1.46; 2.80)	2.27 (1.54; 3.24)	2.24 (1.34; 4.08)	2.03 (1.42; 5.76)	2.17 (1.40; 4.28)
Range [min; max]	[0.50; 4.84]	[1.02; 5.93]	[0.50; 5.93]	[0.49; 5.56]	[0.64; 14.1]	[0.49; 14.1]
*GFAP change from baseline ([pg/mL]/year)*						
Median (Q1; Q3)	91,100 (4950; 141,000)	−41,000 (−83,800; 6500)	14,900 (−31,700; 113,000)	2850 (−1230; 8270)	−843 (−4050; −151)	−352 (−2560; 893)
Range [min; max]	[−28,600; 513,000]	[−149,000; 146,000]	[−149,000; 513,000]	[−11,900; 36,500]	[−31,600; 618]	[−31,600; 36,500]
*Fold change*						
Median (Q1; Q3)	0.49 (−0.03; 3.29)	−0.11 (−0.43; 0.27)	0.08 (−0.19; 0.61)	0.44 (−0.15; 1.09)	−0.41 (−0.51; −0.10)	−0.13 (−0.44; 0.21)
Range [min; max]	[−0.21; 8.70]	[−0.81; 0.60]	[−0.81; 8.70]	[−0.62; 10.1]	[−0.76; 0.34]	[−0.76; 10.1]

## 4 Discussion

GFAP levels in biofluids from individuals with AxD were quantifiable and reproducible using a commercially available assay, with appropriate modifications to the standard recommended protocol. GFAP was elevated in CSF and plasma for all forms of AxD when compared to hospitalized controls and leukodystrophy controls, respectively. GFAP levels were impacted by both patient phenotype (as defined by clinical sub‐type) and age at sample collection with the caveat that disease phenotype is strongly associated with age. However, levels were not stable over time, especially in those from whom sampling began before the age of 8 years. Collectively, these data are a critical initial step in defining biomarker validation and context of use for GFAP in AxD.

The concept that disease severity in AxD is driven by excess GFAP originated from experiments in animal models [16, 17, 18, 19], but whether this is relevant for the human disease remains a point of contention. Only one study examined human tissue, but this was limited to autopsy specimens and only related to end‐stage disease and used age of onset as an indicator for severity [20]. Here we place GFAP values, for both CSF and blood, in the context of the newer classification systems that emphasized either clinical phenotype [8] or anatomical distribution of lesions (the latter combined with clinical phenotype) [9]. We found that GFAP values are elevated in AxD compared to age‐matched samples, irrespective of classification system. In addition, we found that plasma values were uniformly elevated and rank values were distinguishable between groups based on age of onset, Type I and II, and cerebral‐bulbospinal groupings of patients. CSF values were uniformly elevated compared to controls as well but were only distinguishable between the cerebral‐intermediate‐bulbospinal grouping.

We also found that GFAP levels in AxD are not necessarily stable, particularly in those with early onset and evaluated early in their course of disease (i.e., baseline sample collected before the age of 8.2 years for CSF, or 7.9 years for blood). Elevations in GFAP early in the disease is not surprising when viewed in light of previous reports from developmental studies of rodent models. For instance, using the mouse knock‐in that is analogous to the R239H human variant, Jany et al. found that GFAP levels in newborns were indistinguishable at birth but diverged during the first two post‐natal weeks, in part due to increased synthesis due to transactivation of the GFAP promoter [17]. Similarly, using the newer rat model, analogous to the same human variant, Hagemann et al. found a ~10‐fold increase that occurred from 2 to 8 weeks after birth [4]. In both mouse and rat models, GFAP levels rose and then plateaued during early adulthood. The results from our current study therefore fit with this view of how the disease begins and progresses, although we do not yet have sufficient longitudinal data (from either rodents or human) to say what happens after the period of plateau.

### 4.1 Comparison With Other Leukodystrophies

Upregulation of GFAP might be expected for any of the leukodystrophies since this is a general feature of astrocyte response to injury [21]. For instance, increases in CSF and blood levels of GFAP are observed in metachromatic leukodystrophy and X‐linked adrenoleukodystrophy [22, 23]. Here, we directly compared CSF levels of GFAP in AxD with those found in 3 other leukodystrophies: PMD, POLR3‐related, and *TUBB4A*‐related. These hypomyelinating disorders were intentionally selected as we hypothesized that they would involve less astrocytic injury. We found that the AxD samples were consistently and markedly elevated compared to these disorders. We did not have a leukodystrophy control group for the CSF analysis, however, and so cannot determine whether these 3 other leukodystrophies exhibit increased biofluid levels of GFAP.

### 4.2 Context of Use

GFAP concentration in blood has been authorized by the FDA as a diagnostic biomarker in traumatic brain injury [24] and is under investigation for several other disorders as well [10]. Detection of GFAP in plasma predicted those individuals with intracranial lesions on computerized tomography of the brain [25, 26]. GFAP has also been proposed as a predictive biomarker for disability in multiple sclerosis [11], a biomarker for progression in Huntington's disease [27], and a susceptibility/risk biomarker for cognitive decline in Alzheimer's disease [28, 29, 30, 31, 32]. More data are needed to define the context of use in AxD. For example, GFAP concentrations in the CSF and/or plasma might be used as a response biomarker in a therapeutic treatment trial although the clinical relevance to the patient and correlation with clinical efficacy have yet to be established. Alternatively, GFAP data may be used as a diagnostic biomarker in those patients with a GFAP variant but atypical clinical or radiologic features, recognizing that increased GFAP is a relatively non‐specific finding in neurologic disease. Nevertheless, our data provides disease‐specific ranges for comparison of such cases, although age at sample would need to be considered.

### 4.3 Technical Issues

We highlight several important technical issues to consider for future research. First, we highlight the decreasing stability of CSF GFAP when subjected to multiple freeze–thaw cycles. We and others have found that values in blood remain stable through at least 4 freeze–thaws, for both plasma and serum, and with a variety of assays [6, 7, 33, 34, 35, 36, 37, 38]. We note that only Simrén et al. gives sufficient details of their freezing and thawing steps to allow careful replication in other laboratories [36]. In contrast to blood, however, GFAP in CSF does change after freeze–thaws. Simrén et al. reported a 50% decline after 7 cycles using a Simoa assay [36]. We found a more substantial decline after only 4 cycles. While we cannot yet account for the differences between our results and those of Simrén and colleagues, we recommend that CSF samples only be studied after a single freeze‐thaw cycle, and that controls must be handled in the same manner. Second, the values expected for individuals with AxD are higher than in most other conditions and thus warrant higher dilutions than recommended in the manufacturer's instructions. Third, we stress the importance of age‐matching in the selection of controls, particularly for the pediatric population. Three groups have now reported that GFAP in blood is relatively high in newborns but drops substantially over the first 20 years of life [22, 39, 40, 41], and our results are in line with these findings. We also find a similar profile for CSF. Finally, given the high GFAP concentrations and possibility of some intra‐ and inter‐assay variability, testing should include at least 2 aliquots, and longitudinal comparisons should be made only in comparison to a baseline sample run at the same time.

### 4.4 Generalizability to Clinical Care

Although not FDA approved, plasma GFAP tests (that utilize a different methodology than the Simoa platform) are available for clinical care [42, 43]. As such, the GFAP concentrations from a clinical report should not be directly compared to the ranges and figures contained herein. Moreover, our plasma samples were centrifuged and stored in low‐protein binding tubes and longitudinal samples were analyzed at the same time of a baseline sample. In clinical care, samples are sent in standard tubes and analyzed upon receipt. The change in GFAP concentration from day‐to‐day or sample‐to‐sample in clinical testing has not been studied and results should be interpreted with caution as changes may reflect sample or assay variability given the abundance of protein in AxD patients. In addition, age‐related variability, such as an increase in GFAP among young patients and decrease in older patients, may not reflect change in clinical status but rather represent expected age‐related changes.

### 4.5 Limitations

Our study has several limitations. We had small sample sizes, as expected with a rare disease, but especially for those with neonatal onset or providing CSF from ventricular rather than lumbar sites. Using an ELISA assay in a study of 6 individuals with normal pressure hydrocephalus and 2 with primary degenerative dementia (presumed Alzheimer's disease) who provided simultaneous ventricular and lumbar samples, Albrechtsen et al. found that the ventricular values always exceeded the lumbar values [44]. Thus, our ventricular samples were shown in the figures but not included in the tables or analyses. Second, we had limited longitudinal CSF samples, with only 20 (45.45%) of our total 44 participants available for such monitoring and a limit by the IRB on the number of allowable lumbar punctures. Finally, the Simoa assay from Quanterix itself warrants further characterization to define what regions of the GFAP protein are being detected, as the results may be affected by the particulars of different variants present in the patient population, especially those with insertions, deletions, and duplications.

### 4.6 Conclusions

In conclusion, our study provides guidelines for sample handling and assay protocols that are specific to AxD and presents a comprehensive picture of GFAP values that can be expected for individuals with several *GFAP* variants, different clinical phenotypes, and at various stages of their disease. These values can serve as an important foundation for interpreting changes over time in patient populations, although with the current state of knowledge and assay validation challenges, GFAP by itself is not ready for use in assessing disease status or progression in AxD. However, GFAP may prove useful for future studies in combination with other biomarkers such as neurofilament light and phosphorylated forms of tau [7], or alongside other clinical measures such as MRI and functional assessments. Additionally, when considering the limitations of CSF collection, the utility of plasma as an appropriate and accessible medium for data collection of peripheral GFAP levels could be further explored.

## Author Contributions

Study conception and design (A.T.W., A.T., A.L.V.), data acquisition (J.Y.J., G.W.L., S.N.), data analysis (K.A., A.P., W.F., S.W.), data interpretation (A.T.W., A.T., J.Y.J., G.W.L., K.A., A.P., W.F., S.W., S.N., A.L.V.), drafting the manuscript (A.T.W., A.T., S.W.), manuscript review for intellectual content (J.Y.J., G.W.L., K.A., A.P., W.F., S.N., A.L.V.), final approval (A.T.W., A.T., J.Y.J., G.W.L., K.A., A.P., W.F., S.W., S.N., A.L.V.), accountability (A.T.W., A.T., J.Y.J., G.W.L., K.A., A.P., W.F., S.W., S.N., A.L.V.).

## Funding

This work was funded by the NIH (U54NS115052), Ionis Pharmaceuticals, Elise's Corner, and Grayson's Ladder.

## Conflicts of Interest

A.T.W. has received grant support from the NIH (NINDS) (U54NS115052, co‐lead investigator, subproject); research support from Ionis Pharmaceuticals (Investigator‐initiated research), Ionis Pharmaceuticals (Clinical trial support—Alexander disease and Pelizaeus Merzbacher disease), Roche/Genentech (Clinical trial support), Novartis (Clinical trial support), PassageBio (Clinical trial support), Sarepta (Clinical trial support), Pfizer (Clinical trial support); personal compensation for serving on a data safety monitoring board (SwanBio); and publishing royalties (UpToDate, MedLink Neurology). A.L.V. receives grants, clinical trial support, and in‐kind support for research from Eli Lilly and Company, Gilead Sciences, Takeda Pharmaceuticals, Illumina, Biogen, Homology Medicines, Ionis Pharmaceuticals, Passage Bio, Orchard Therapeutics, Sana Pharmaceuticals, SynaptixBio, Boehringer Ingelheim, Myrtelle, and Bluebird Bio. A.L.V. serves on the scientific advisory boards of the European Leukodystrophy Association and the United Leukodystrophy Foundation, as well as in an unpaid capacity for Takeda Pharmaceuticals, Ionis, Biogen, and Illumina. A.L.V. has received support for attending meetings from the European Leukodystrophy Association, the United Leukodystrophy Foundation, and the Recordati Rare Diseases Foundation. A.L.V. has planned, issued, or pending patents in a TUBB4A antisense oligonucleotide and ISGs as a tool in AGS. The AGS scale is copyrighted by the Children's Hospital of Philadelphia. The other authors declare no conflicts of interest.

## Supporting information


**Figure S1:** Serial dilution curves for determination of AxD sample dilutions. Dilution curves were created for 15 AxD participants' samples (10 CSF and 10 plasma samples). Dashed lines of the same color represent a duplicate run of the same sample. Samples with greater concentrations of GFAP frequently did not have a detectable result using the assay, represented by open circles (at that dilution factor, the concentration was above the limit of detection). The dilution factors that resulted in detectable levels for every AxD patient were 1:1600 for CSF and 1:160 for plasma.
**Table S1:** Intra‐assay variability in serial dilution curves. Dilutions of 1:1600 (CSF) and 1:160 (Plasma) were the first dilutions to have no samples ALQ and above 10% CV. [ALQ, above the limit of quantification; CV, coefficient of variance; NA, not applicable (samples out of range did not yield a concentration for comparison of the coefficient of variance), *N*, number of samples; SD, standard deviation].
**Table S2:** Fold Change Summary of Freeze/Thaw Effects on GFAP Concentration in CSF and Plasma.
**Table S3:** Intra‐assay variability (CV %) in CSF and plasma samples by group.
**Table S4:** Inter‐assay variability (CV %) in CSF and plasma samples by group.
**Table S5:** Cohort description at baseline (excluding samples with CV > 10% or single replicate value).
**Figure S2:** Freeze/Thaw Effects on GFAP Concentrations in AxD CSF and Plasma. (A) GFAP concentration declines in CSF after a single freeze and thaw cycle and continues to decline with each cycle. The mean fold change was 0.71 per thaw in CSF. (B) GFAP concentrations in plasma remained stable after a single thaw (mean fold change 0.90). See Table S2 for the summarized fold change across the samples and thaw cycles.
**Figure S3:** Sample variability in CSF. Four aliquots (run in duplicate) were evaluated within plate and between plates for 6 subjects. (A) *Bland–Altman plot of 24 replicates GFAP concentrations*. The mean difference in concentration between replicate 2 and replicate 1 was 1.9 pg/mL (95% CI −39.5 to 43.3, SD 21.1, median 4.7, range −46.7 to 47.3). The mean absolute difference in concentration was 14.4 pg/mL (SD 15.2 pg/mL) with a median of 7.7 pg/mL (range 1.98–47.3 pg/mL). The CV of the replicates ranged from 0.0% to 8.0% with a median of 2.0%, all < 10% and within the typical range for intra‐assay variability. (B) *Intra‐assay variability between aliquots*. The difference in mean values between aliquots within the same plate was on average 35.6 pg/mL (SD 38.6 pg/mL, median 22.2 pg/mL, range 0.89–136.8 pg/mL). The within plate CV ranged from 1.05% to 15.9% with a median of 7.24% (3 samples > 10% in run 2, max 15.9%). When comparing the concentrations between aliquots within runs the values were not significantly different between aliquots (Wilcoxon signed rank test: run 1 *p* = 0.0625, run 2 *p* = 0.688). (C) Inter‐assay variability between aliquots: The difference in plate means between runs was on average 54.2 pg/mL (SD 48.8 pg/mL, median 42.2 pg/mL, range 10.1–136.6 pg/mL). The within plate CV ranged from 3.6% to 16.2% with a median of 11.7% (1 sample above 15%, max 16.2%). When comparing the plate means the values were not significantly different between runs (Wilcoxon signed rank test: *p* = 0.438).
**Figure S4:** Baseline GFAP levels across recurrent GFAP variants. (A) Baseline GFAP concentration was not significantly different across the recurrent variants (Kruskal‐Wallis test: CSF, *p* = 0.1710; plasma, *p* = 0.1110). (B) Baseline age was significantly different across recurrent variant groups for plasma (Kruskal‐Wallis test, CSF: *p* = 0.2150, plasma: *p* = 0.0042). Group sample sizes were small for CSF, in particular.
**Table S6:** GFAP variants reported in the Alexander disease cohort. *Variants are aligned with the MANE transcript (NM_002055.5) based on the transcript reported in the testing laboratory report, if available. For cases with no report or no transcript listed, the variant is checked using the date of variant discovery, if known, NCBI/Ensembl, and a comparison of nucleotide and amino acid changes to identify the most likely transcript.
**Variant classification is according to the pathogenicity on the original laboratory report.
***This variant was reported by the laboratory. It was subsequently acknowledged that it was an error, although a correction was never provided. Thus, the second variant could not be verified.

## Data Availability

The data that support the findings of this study are available from the corresponding author upon reasonable request.
