# Supplementary material for: Characterization of Clinical Phenotype to Glial Fibrillary Acidic Protein Concentrations in Alexander Disease
Source: Ann Clin Transl Neurol. 2026 Jan 9;13(6):1206–21. doi: 10.1002/acn3.70305 (PMC13251449; doi:10.1002/acn3.70305)
Supplement: Supplementary file 1 — Figure S1: Serial dilution curves for determination of AxD sample dilutions. Dilution curves were created for 15 AxD participants' samples (10 CSF and 10 plasma samples). Dashed lines of the same color represent a duplicate run of the same sample. Samples with greater concentrations of GFAP frequently did not have a detectable result using the assay, represented by open circles (at that dilution factor, the concentration was above the limit of detection). The dilution factors that resulted in detectable levels for every AxD patient were 1:1600 for CSF and 1:160 for plasma. Table S1: Intra‐assay variability in serial dilution curves. Dilutions of 1:1600 (CSF) and 1:160 (Plasma) were the first dilutions to have no samples ALQ and above 10% CV. [ALQ, above the limit of quantification; CV, coefficient of variance; NA, not applicable (samples out of range did not yield a concentration for comparison of the coefficient of variance), N, number of samples; SD, standard deviation]. Table S2: Fold Change Summary of Freeze/Thaw Effects on GFAP Concentration in CSF and Plasma. Table S3: Intra‐assay variability (CV %) in CSF and plasma samples by group. Table S4: Inter‐assay variability (CV %) in CSF and plasma samples by group. Table S5: Cohort description at baseline (excluding samples with CV > 10% or single replicate value). Figure S2: Freeze/Thaw Effects on GFAP Concentrations in AxD CSF and Plasma. (A) GFAP concentration declines in CSF after a single freeze and thaw cycle and continues to decline with each cycle. The mean fold change was 0.71 per thaw in CSF. (B) GFAP concentrations in plasma remained stable after a single thaw (mean fold change 0.90). See Table S2 for the summarized fold change across the samples and thaw cycles. Figure S3: Sample variability in CSF. Four aliquots (run in duplicate) were evaluated within plate and between plates for 6 subjects. (A) Bland–Altman plot of 24 replicates GFAP concentrations. The mean difference in concentration between replic [file ACN3-13-1206-s001.docx]

**Characterization of Clinical Phenotype to Glial Fibrillary Acidic Protein Concentrations in Alexander Disease**

**Supplemental Data**

Amy T. Waldman, MD, MSCE^1,2^, Asako Takanohashi, PhD, DVM^1^, Joshua Y. Joung, MS^1^, Geraldine W. Liu, MA^1^, Kaley Arnold, MS, LCGC^1^, Amy Pizzino, MS, CGC^1^, Walter Faig, PhD ^3^, Sarah Woidill, MS^1^, Sona Narula, MD^1,2^, Adeline L. Vanderver, MD^1,2^

^1^Division of Neurology, Children’s Hospital of Philadelphia, Philadelphia, PA

^2^Department of Neurology, Perelman School of Medicine at the University of Pennsylvania, Philadelphia, PA

^3^Biostatistics and Data Management Core, Children’s Hospital of Philadelphia, Philadelphia, PA

**Corresponding Author:**

Amy T. Waldman, MD, MSCE

Children’s Hospital of Philadelphia

The Hub for Clinical Collaboration

Division of Neurology, 10^th^ floor, Room 10524

3500 Civic Center Boulevard

Philadelphia, PA 19104

[waldman@chop.edu](mailto:waldman@chop.edu)

**Supplemental Figure 1: Serial Dilution Curves for Determination of AxD Sample Dilutions.**
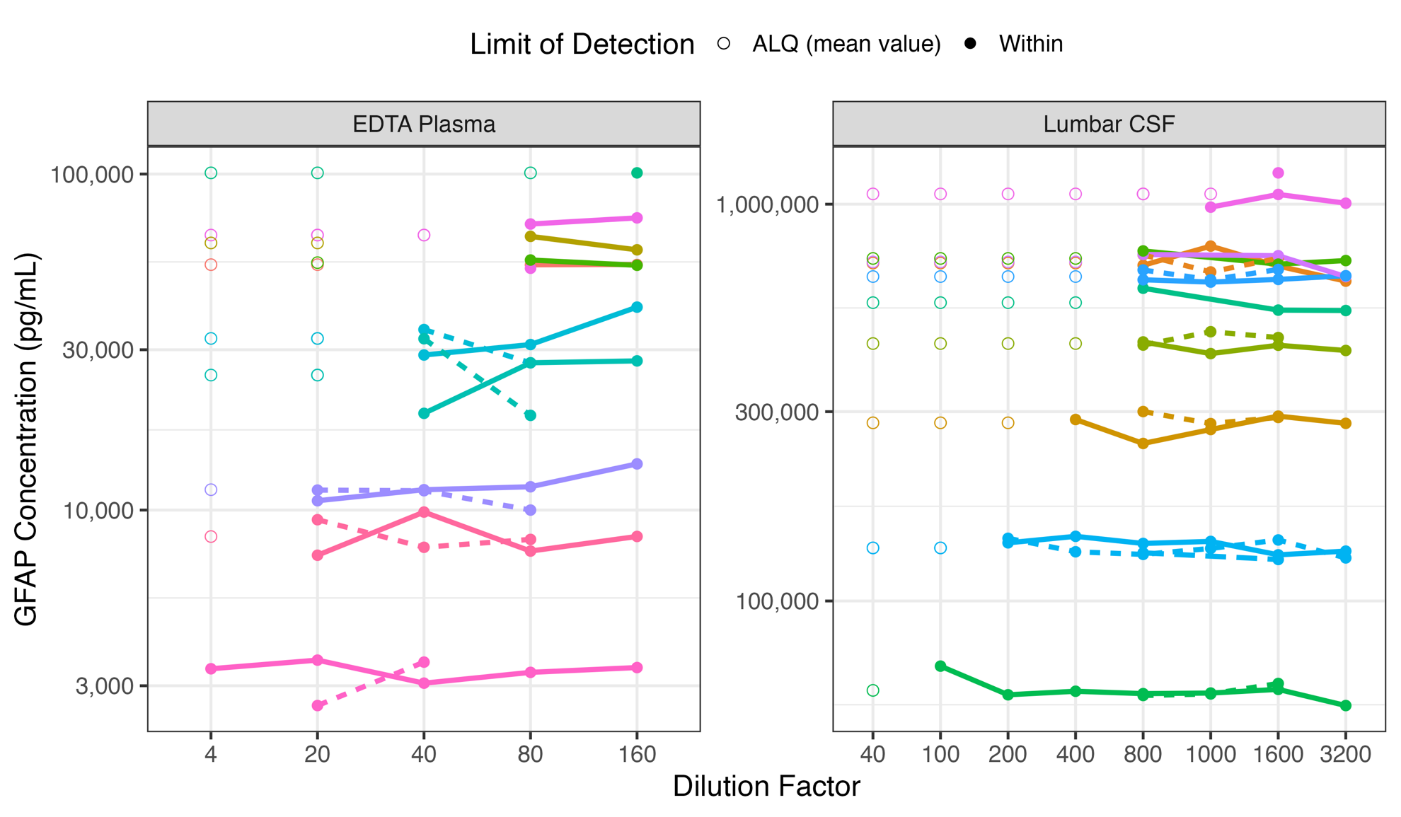


**Supplemental Table 1: Intra-assay variability in serial dilution curves**.

| **Sample type** | **Dilution** | **Mean CV, % (SD)** | **Samples tested (N)** | **Samples ALQ (N)** | **Samples ALQ (%)** | **CV >10% (N)** | **CV >10% (%)** |
| --- | --- | --- | --- | --- | --- | --- | --- |
| CSF | 40 | NaN (NA) | 11 | 11 | 100 | 0 | 0 |
| CSF | 100 | 4.9 (NA) | 11 | 10 | 90.91 | 0 | 0 |
| CSF | 200 | 2.26 (0.86) | 11 | 8 | 72.73 | 0 | 0 |
| CSF | 400 | 1.23 (1.77) | 11 | 7 | 63.64 | 0 | 0 |
| CSF | 800 | 2.91 (2.19) | 18 | 3 | 16.67 | 0 | 0 |
| CSF | 1000 | 2.88 (2.47) | 14 | 2 | 14.29 | 0 | 0 |
| CSF | 1600 | 3.06 (2.07) | 18 | 0 | 0 | 0 | 0 |
| CSF | 3200 | 2.37 (2.15) | 11 | 0 | 0 | 0 | 0 |
| plasma | 4 | 2.23 (NA) | 10 | 9 | 90 | 0 | 0 |
| plasma | 20 | 6.17 (4.91) | 16 | 10 | 62.5 | 2 | 12.5 |
| plasma | 40 | 3.77 (2.83) | 12 | 2 | 16.67 | 0 | 0 |
| plasma | 80 | 3.05 (1.75) | 15 | 1 | 6.67 | 0 | 0 |
| plasma | 160 | 3.13 (1.93) | 10 | 0 | 0 | 0 | 0 |

**Supplemental Figure 2: Freeze/Thaw Effects on GFAP Concentrations in AxD CSF and Plasma.**

| **A CSF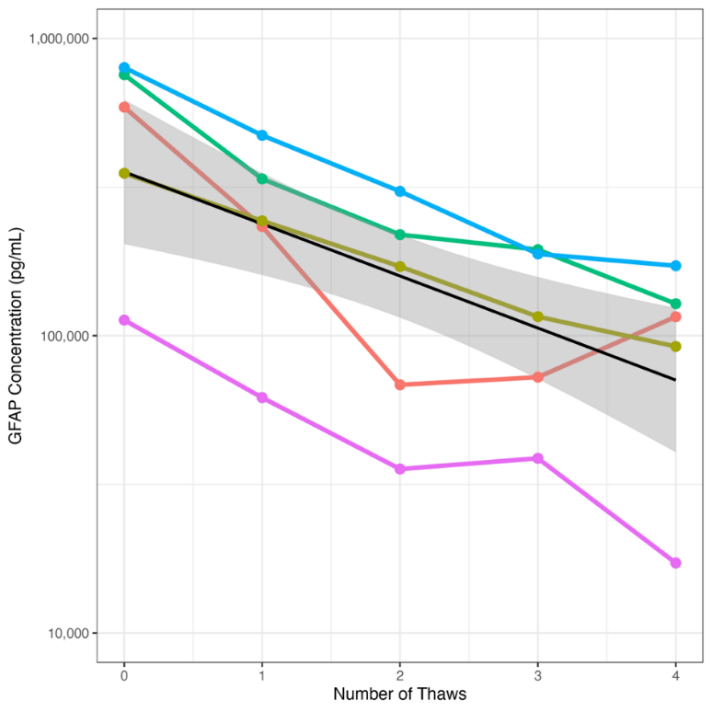** | **B Plasma**  **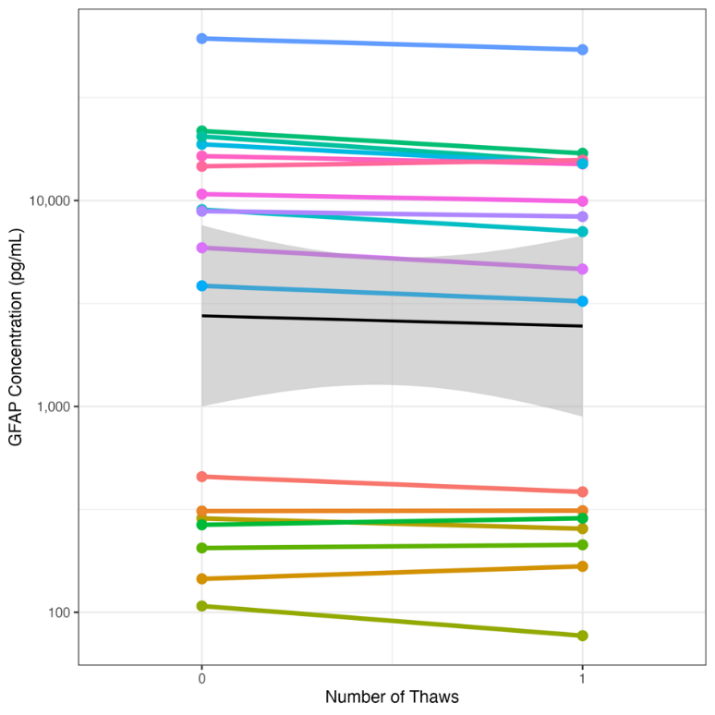** |
| --- | --- |

**Supplemental Table 2: Fold Change Summary of Freeze/Thaw Effects on GFAP Concentration in CSF and Plasma.**

| **Sample Medium** | **Thaw #** | **N** | **Fold Change (sequential)** | | | | | | **Fold Change (from original)** | | | | | |
| --- | --- | --- | --- | --- | --- | --- | --- | --- | --- | --- | --- | --- | --- | --- |
|  |  |  | **Min** | **Max** | **Mean** | **SD** | **Median** | **IQR** | **Min** | **Max** | **Mean** | **SD** | **Median** | **IQR** |
| **Lumbar CSF** | 1 | 5 | 0.40 | 0.69 | 0.54 | 0.12 | 0.55 | 0.15 | 0.40 | 0.69 | 0.54 | 0.12 | 0.55 | 0.15 |
|  | 2 | 5 | 0.29 | 0.70 | 0.57 | 0.16 | 0.65 | 0.07 | 0.12 | 0.49 | 0.32 | 0.14 | 0.32 | 0.09 |
|  | 3 | 5 | 0.62 | 1.09 | 0.87 | 0.21 | 0.89 | 0.38 | 0.12 | 0.34 | 0.26 | 0.09 | 0.26 | 0.09 |
|  | 4 | 5 | 0.44 | 1.60 | 0.88 | 0.44 | 0.79 | 0.25 | 0.15 | 0.26 | 0.20 | 0.04 | 0.20 | 0.05 |
| **EDTA Plasma** | 1 | 18 | 0.72 | 1.15 | 0.90 | 0.13 | 0.89 | 0.20 |  |  |  |  |  |  |

**Supplemental Table 3: Intra-assay variability (CV%) in CSF and Plasma Samples by Group.**

| **Sample Medium** | **Sample Group** | **N** | **Min CV%** | **Max CV%** | **Mean CV%** | **SD CV%** | **Median CV%** | **IQR CV%** |
| --- | --- | --- | --- | --- | --- | --- | --- | --- |
| **Lumbar CSF** | Alexander Disease | 69 | 0.16 | 8.68 | 2.37 | 1.86 | 2.17 | 2.37 |
|  | Hospital Controls | 46 | 0.13 | 23.06 | 3.00 | 4.10 | 1.92 | 2.39 |
|  | Overall | 115 | 0.13 | 23.06 | 2.62 | 2.97 | 1.98 | 2.41 |
| **EDTA Plasma** | Alexander Disease | 187 | 0.01 | 34.53 | 3.17 | 3.64 | 2.40 | 2.81 |
|  | Leukodystrophy Controls | 59 | 0.13 | 22.30 | 8.01 | 6.42 | 5.95 | 11.07 |
|  | Overall | 246 | 0.01 | 34.53 | 4.33 | 4.91 | 2.89 | 3.93 |

**Supplemental Table 4: Inter-assay variability (CV%) in CSF and Plasma Samples by Group.**

| **Sample Medium** | **Sample Group** | **N** | **Min CV%** | **Max CV%** | **Mean CV%** | **SD CV%** | **Median CV%** | **IQR CV%** |
| --- | --- | --- | --- | --- | --- | --- | --- | --- |
| **Lumbar CSF** | Alexander Disease | 13 | 2.41 | 35.38 | 16.36 | 10.12 | 13.86 | 18.69 |
| **EDTA Plasma** | Alexander Disease | 45 | 0.25 | 32.48 | 9.85 | 7.79 | 6.34 | 11.54 |
|  | Leukodystrophy Controls | 10 | 3.06 | 20.19 | 10.29 | 6.84 | 7.59 | 12.00 |
|  | Overall | 55 | 0.25 | 32.48 | 9.93 | 7.57 | 6.55 | 11.60 |

**Supplemental Table 5: Cohort Description at Baseline (excluding samples with CV > 10% or single replicate value).**

|  | **Lumbar CSF** | | | **EDTA Plasma** | | |
| --- | --- | --- | --- | --- | --- | --- |
|  | **Alexander Disease** | **Hospital Controls** | ***P-*value** | **Alexander Disease** | **Leukodystrophy Controls** | ***P-*value** |
|  | N=44 | N=44 |  | N=91 | N=39 |  |
| Sex (%) | | | | | | |
| Female | 20 (45.5%) | 18 (40.9%) | 0.8296 | 42 (46.2%) | 16 (41.0%) | 0.7290 |
| Male | 24 (54.5%) | 26 (59.1%) |  | 49 (53.8%) | 23 (59.0%) |  |
| Age at sampling (years) | | | | | | |
| Median  (Q1; Q3) | 7.17  (3.56; 11.7) | 7.00  (4.00; 12.0) | 0.9169 | 8.84  (2.93; 17.1) | 9.34  (4.07; 17.1) | 0.7819 |
| Range  [Min; Max] | [0.97; 30.8] | [0.83; 18.0] |  | [0.10; 68.3] | [0.71; 37.5] |  |
| Baseline GFAP Concentration (pg/mL) | | | | | | |
| Median  (Q1; Q3) | 303,000  (122,000; 507,000) | 3,810  (2,410; 5,970) | <0.0001 | 15,600  (4,630; 28,600) | 278  (208; 457) | <0.0001 |
| Range  [Min; Max] | [48,300; 1,070,000] | [33.7; 19,100] |  | [345; 103,000] | [48.2; 910] |  |

**Supplemental Figure 3: Sample variability in CSF.**

| **A**  **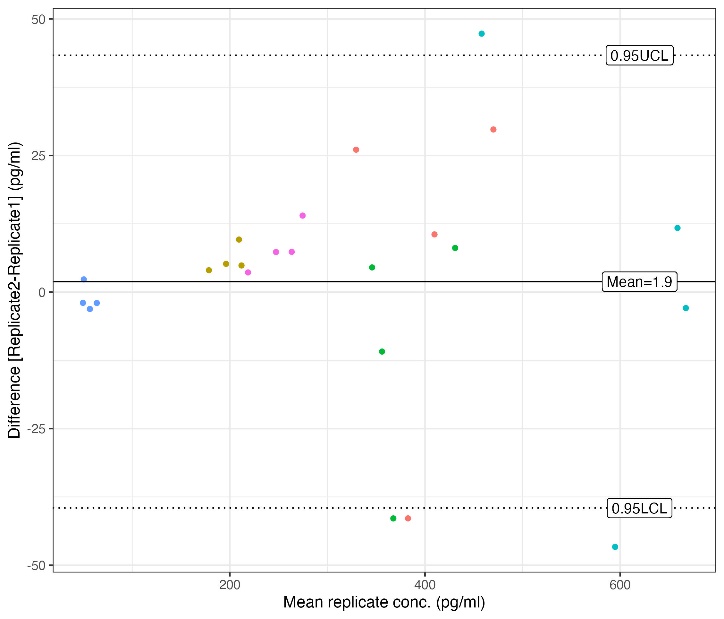** | **B**  **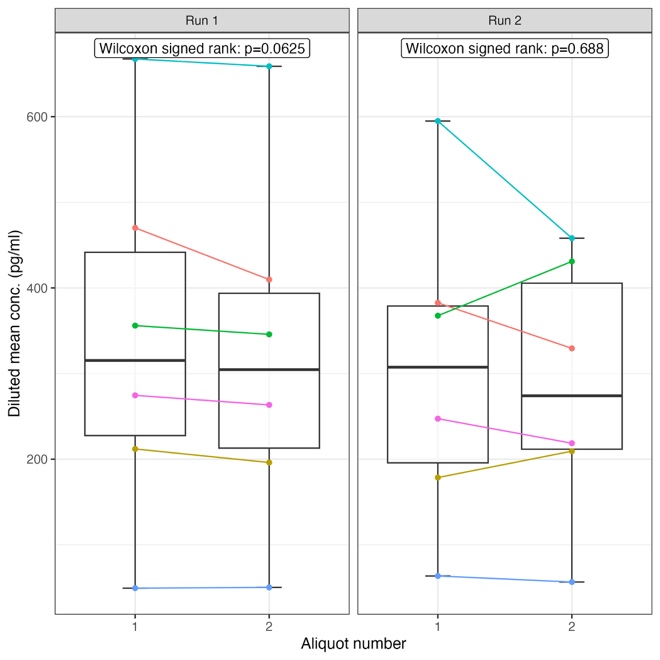** |
| --- | --- |
| **C**  **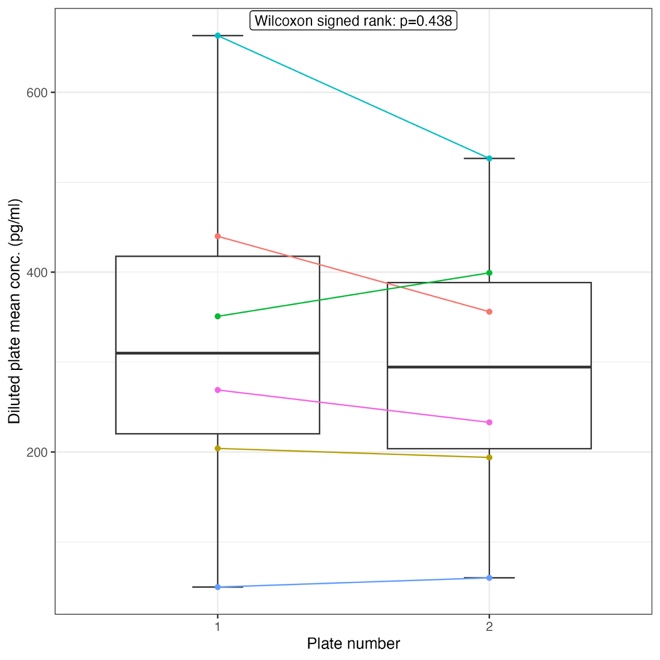** |  |

**Supplemental Figure 4: Baseline GFAP Levels Across Recurrent GFAP Variants.**

| **A**  **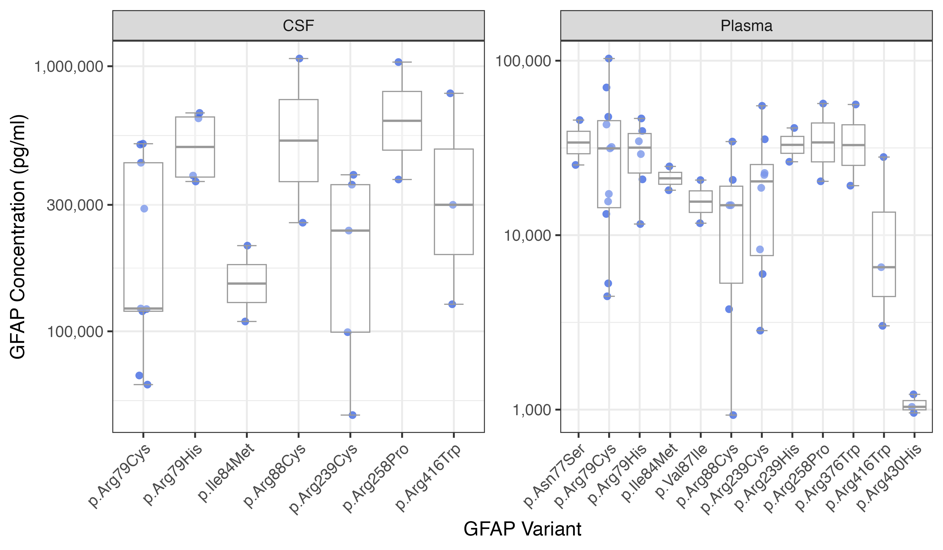** |
| --- |
| **B**  **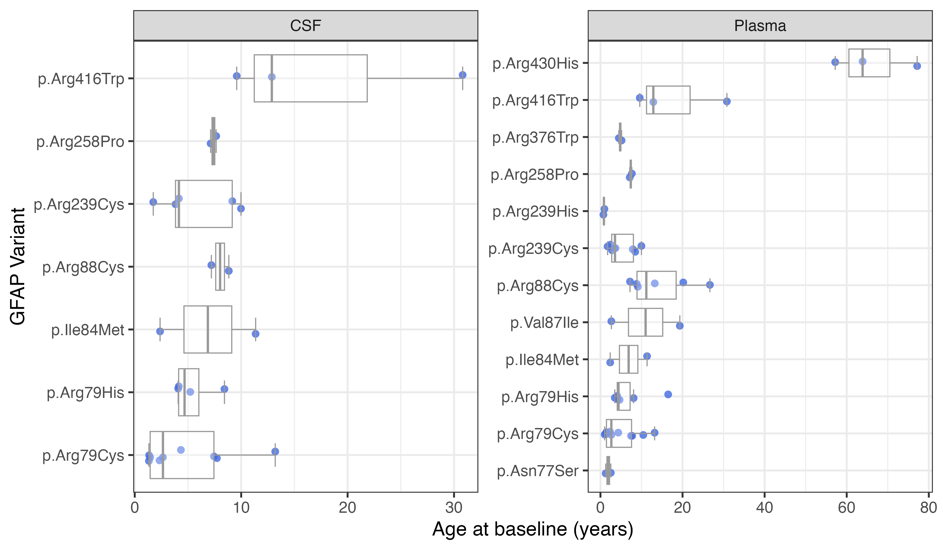** |

**Supplemental Table 6: GFAP variants reported in the Alexander Disease cohort.**

| **AxD Subject** | **Variant 1 nucleotide change*** | **Variant 1 amino acid change*** | **Variant 1 classification**** | **Variant 2 nucleotide change*** | **Variant 2 amino acid change*** | **Variant 2 classification**** |
| --- | --- | --- | --- | --- | --- | --- |
| 1 | c.256A>G | p.Lys86Glu | Likely Pathogenic |  |  |  |
| 2 | c.262C>T | p.Arg88Cys | Pathogenic |  |  |  |
| 3 | c.262C>T | p.Arg88Cys | Pathogenic |  |  |  |
| 4 | c.230A>G | p.Asn77Ser | Pathogenic | Error*** | p.Ser152Leu*** | Uncertain Significance |
| 5 | c.868C>G | p.Gln290Glu | Likely Pathogenic |  |  |  |
| 6 | c.715C>T | p.Arg239Cys | Pathogenic |  |  |  |
| 7 | c.368T>C | p.Leu123Pro | Uncertain Significance |  |  |  |
| 8 | c.1250A>C | p.Asp417Ala | Likely Pathogenic |  |  |  |
| 9 | c.1250A>C | p.Asp417Ala | Likely Pathogenic |  |  |  |
| 10 | c.1250A>C | p.Asp417Ala | Likely Pathogenic |  |  |  |
| 11 | c.382G>A | p.Asp128Asn | Uncertain Significance |  |  |  |
| 12 | c.227T>A | p.Leu76His | Uncertain Significance | c.228C>A | p.Leu76= | Uncertain Significance |
| 13 | c.1084G>A | p.Glu362Lys | Likely Pathogenic |  |  |  |
| 14 | c.1171+473C>A |  | Uncertain Significance |  |  |  |
| 15 | c.1171+473C>A |  | Uncertain Significance |  |  |  |
| 16 | c.715C>T | p.Arg239Cys | Pathogenic |  |  |  |
| 17 | [c.1171+472G>A](https://www.ncbi.nlm.nih.gov/nuccore/NM_002055.5?report=graph&search=NM_002055.5%3Ac.1171%2B472G%3EA) |  | Uncertain Significance |  |  |  |
| 18 | [c.1171+472G>A](https://www.ncbi.nlm.nih.gov/nuccore/NM_002055.5?report=graph&search=NM_002055.5%3Ac.1171%2B472G%3EA) |  | Uncertain Significance |  |  |  |
| 19 | c.236G>A | p.Arg79His | Pathogenic |  |  |  |
| 20 | c.1171+472G>A |  | Uncertain Significance |  |  |  |
| 21 | c.1078G>C | p.Asp360His | Pathogenic |  |  |  |
| 22 | c.236G>A | p.Arg79His | Pathogenic |  |  |  |
| 23 | c.715C>T | p.Arg239Cys | Pathogenic |  |  |  |
| 24 | c.835A>G | p.Lys279Glu | Likely Pathogenic |  |  |  |
| 25 | c.773G>C | p.Arg258Pro | Pathogenic |  |  |  |
| 26 | c.262C>T | p.Arg88Cys | Pathogenic |  |  |  |
| 27 | c.259G>A | p.Val87Ile | Pathogenic |  |  |  |
| 28 | c.263G>T | p.Arg88Leu | Uncertain Significance | c.469G>A | p.Asp157Asn | Benign |
| 29 | c.236G>A | p.Arg79His | Pathogenic |  |  |  |
| 30 | c.1111G>C | p.Glu371Gln | Pathogenic |  |  |  |
| 31 | c.235C>T | p.Arg79Cys | Pathogenic |  |  |  |
| 32 | c.1246C>T | p.Arg416Trp | Pathogenic |  |  |  |
| 33 | c.682A>G | p.Lys228Glu | Uncertain Significance |  |  |  |
| 34 | c.252C>G | p.Ile84Met | Likely Pathogenic |  |  |  |
| 35 | c.1246C>T | p.Arg416Trp | Likely Pathogenic |  |  |  |
| 36 | c.940G>A | p.Glu314Lys | Likely Pathogenic |  |  |  |
| 37 | c.235C>T | p.Arg79Cys | Pathogenic |  |  |  |
| 38 | c.232G>T | p.Asp78Tyr | Pathogenic |  |  |  |
| 39 | c.232G>T | p.Asp78Tyr | Pathogenic |  |  |  |
| 40 | c.235C>T | p.Arg79Cys | Pathogenic |  |  |  |
| 41 | c.1220A>C | p.Asn407Thr | Uncertain Significance |  |  |  |
| 42 | c.713T>A | p.Ile238Asn | Likely Pathogenic |  |  |  |
| 43 | c.252C>G | p.Ile84Met | Uncertain Significance |  |  |  |
| 44 | c.620A>T | p.Glu207Val | Likely Pathogenic |  |  |  |
| 45 | c.235C>T | p.Arg79Cys | Pathogenic |  |  |  |
| 46 | c.715C>G | p.Arg239Gly | Likely Pathogenic |  |  |  |
| 47 | c.1138C>T | p.Pro380Ser | Pathogenic |  |  |  |
| 48 | c.235C>T | p.Arg79Cys | Pathogenic |  |  |  |
| 49 | c.1105C>G | p.Leu369Val | Uncertain Significance |  |  |  |
| 50 | c.773G>C | p.Arg258Pro | Pathogenic |  |  |  |
| 51 | c.1126C>T | p.Arg376Trp | Pathogenic |  |  |  |
| 52 | c.739T>C | p.Ser247Pro | Pathogenic |  |  |  |
| 53 | c.715C>T | p.Arg239Cys | Pathogenic |  |  |  |
| 54 | c.1112A>C | p.Glu371Ala | Uncertain Significance |  |  |  |
| 55 | c.236G>A | p.Arg79His | Pathogenic |  |  |  |
| 56 | c.262C>T | p.Arg88Cys | Pathogenic |  |  |  |
| 57 | c.235C>T | p.Arg79Cys | Pathogenic |  |  |  |
| 58 | c.262C>T | p.Arg88Cys | Pathogenic |  |  |  |
| 59 | c.197G>A | p.Arg66Gln | Likely Pathogenic |  |  |  |
| 60 | c.715C>T | p.Arg239Cys | Pathogenic | c.1258-1G>T | p. ? | Uncertain Significance |
| 61 | c.235C>T | p.Arg79Cys | Pathogenic |  |  |  |
| 62 | c.1220A>C | p.Asn407Thr | Uncertain Significance |  |  |  |
| 63 | c.375_380dup | p.Arg126_Leu127dup | Pathogenic |  |  |  |
| 64 | c.715C>T | p.Arg239Cys | Pathogenic |  |  |  |
| 65 | c.269T>C | p.Leu90Pro | Pathogenic |  |  |  |
| 66 | c.262C>T | p.Arg88Cys | Pathogenic |  |  |  |
| 67 | c.1084G>A | p.Glu362Lys | Pathogenic |  |  |  |
| 68 | c.235C>T | p.Arg79Cys | Pathogenic |  |  |  |
| 69 | c.235C>T | p.Arg79Cys | Pathogenic |  |  |  |
| 70 | c.231T>A | p.Asn77Lys | Pathogenic |  |  |  |
| 71 | c.1246C>T | p.Arg416Trp | Pathogenic |  |  |  |
| 72 | c.716G>A | p.Arg239His | Pathogenic |  |  |  |
| 73 | c.236G>A | p.Arg79His | Pathogenic |  |  |  |
| 74 | c.236G>A | p.Arg79His | Pathogenic |  |  |  |
| 75 | c.259G>A | p.Val87Ile | Pathogenic |  |  |  |
| 76 | c.1111G>A | p.Glu371Lys | Pathogenic |  |  |  |
| 77 | c.1049A>G | p.Gln350Arg | Uncertain Significance |  |  |  |
| 78 | c.230A>G | p.Asn77Ser | Pathogenic |  |  |  |
| 79 | c.715C>T | p.Arg239Cys | Pathogenic |  |  |  |
| 80 | c.235C>T | p.Arg79Cys | Pathogenic |  |  |  |
| 81 | c.716G>A | p.Arg239His | Pathogenic |  |  |  |
| 82 | c.1046A>G | p.Tyr349Cys | Uncertain Significance |  |  |  |
| 83 | c.715C>T | p.Arg239Cys | Pathogenic |  |  |  |
| 84 | c.731C>T | p.Ala244Val | Pathogenic |  |  |  |
| 85 | c.708_710del | p.Glu237del | Likely Pathogenic |  |  |  |
| 86 | c.1126C>G | p.Arg376Gly | Likely Pathogenic |  |  |  |
| 87 | c.389T>C | p.Leu130Pro | Likely Pathogenic |  |  |  |
| 88 | c.235C>T | p.Arg79Cys | Pathogenic |  |  |  |
| 89 | c.1126C>T | p.Arg376Trp | Pathogenic |  |  |  |
| 90 | c.769T>G | p.Tyr257Asp | Uncertain Significance |  |  |  |
| 91 | c.235C>T | p.Arg79Cys | Pathogenic |  |  |  |
| 92 | c.230A>G | p.Asn77Ser | Pathogenic |  |  |  |
| 93 | c.617_618+1del | p.? | Pathogenic |  |  |  |
| 94 | c.802G>C | p.Ala268Pro | Likely Pathogenic |  |  |  |
| 95 | c.259G>A | p.Val87Ile | Pathogenic |  |  |  |
| 96 | c.677T>A | p.Val226Glu | Unknown/Not Reported |  |  |  |
